# Complete Genome Sequence of *Spiroplasma apis* B31^T^ (ATCC 33834), a Bacterium Associated with May Disease of Honeybees (*Apis mellifera*)

**DOI:** 10.1128/genomeA.01151-13

**Published:** 2014-01-09

**Authors:** Chuan Ku, Wen-Sui Lo, Ling-Ling Chen, Chih-Horng Kuo

**Affiliations:** Institute of Plant and Microbial Biology, Academia Sinica, Taipei, Taiwana; Molecular and Biological Agricultural Sciences Program, Taiwan International Graduate Program, National Chung Hsing University and Academia Sinica, Taipei, Taiwanb; Graduate Institute of Biotechnology, National Chung Hsing University, Taichung, Taiwanc; Biotechnology Center, National Chung Hsing University, Taichung, Taiwand

## Abstract

*Spiroplasma apis* B31^T^ (ATCC 33834) is a wall-less bacterium in the class *Mollicutes* that has been linked to May disease of honeybees (*Apis mellifera*). Here, we report the complete genome sequence of this bacterium to facilitate the investigation of its virulence factors.

## GENOME ANNOUNCEMENT

The bacterial strain *Spiroplasma apis* B31^T^ was isolated from a honeybee (*Apis mellifera*) in southwestern France affected by May disease (1). Previous experiments demonstrated that the artificial infection of honeybees by *S. apis* through injection or food ingestion leads to classical symptoms of May disease, such as nervous disorder, loss of flight ability, and eventually, high mortality in a few days (2). Moreover, high titers of *S. apis* cells were found in the hemolymph of the dead bees. The lethal effect of *S. apis* infection on bees might be prevented by tetracycline, but not by penicillin (2). To investigate the virulence factors of *S. apis* and to improve the taxon sampling of available *Spiroplasma* sequences for comparative analysis, we obtained *S. apis* B31^T^ from the American Type Culture Collection (ATCC 33834) for complete genome sequencing.

The procedures for genome sequencing, assembly, and annotation are based on those described in our previous studies (3, 4). Briefly, the Illumina HiSeq 2000 platform was used to generate 101-bp reads from one paired-end library (~160-bp insert, 6,146,318 reads) and one mate-pair library (~3.8-kb insert, 8,552,782 reads). The *de novo* assembly was performed using ALLPATHS-LG release 42781 (5). The initial assembly was iteratively improved by mapping the raw reads to the contigs by the Burrows-Wheeler Aligner (BWA) version 0.6.2 (6) for visual inspection by IGV version 2.1.24 (7). All gaps were filled by using reads overhanging at the contig margins or by reassembling the reads mapped to the gap regions with Phrap version 1.090518 (http://www.phrap.org/). The programs RNAmmer (8), tRNAscan-SE (9), and Prodigal (10) were used for gene prediction. For each protein-encoding gene, the gene name and product description were annotated based on the orthologous genes identified by OrthoMCL (11) in other *Spiroplasma* genomes (4, 12, 13), including those of *S. chrysopicola* (GenBank accession no. CP005077), *S. diminutum* (GenBank accession no. CP005076), *S. melliferum* (GenBank accession no. AMGI01000001 to AMGI01000024), *S. syrphidicola* (GenBank accession no. CP005078), and *S. taiwanense* (GenBank accession no. CP005074 to CP005075). The genes that do not have identifiable orthologs in other *Spiroplasma* genomes were manually curated based on BLASTp (14) searches against the NCBI nonredundant (nr) protein database (15). The Kyoto Encyclopedia of Genes and Genomes (KEGG) database (16) was used to assist annotation.

The circular chromosome of *S. apis* B31^T^ is 1,160,554 bp in size, with a 28.3% G+C content, and no plasmid was found. The first version of annotation includes one set of 16S-23S-5S rRNA genes, 29 tRNA genes (covering all 20 amino acids), and 997 protein-encoding genes.

### Nucleotide sequence accession number.

The complete genome sequence of *S. apis* B31^T^ has been deposited at DDBJ/EMBL/GenBank under the accession no. CP006682.
